# High-Intensity Interval Training Minimizes the Deleterious Effects of Arterial Hypertension on the Urinary Bladder of Spontaneously Hypertensive Rats

**DOI:** 10.1155/2023/9979397

**Published:** 2023-02-21

**Authors:** Victor Rogério Garcia Batista, Rafael Ribeiro Correia, Vítor S. Fernandes, Allice Santos Cruz Veras, Maria Eduarda Almeida Tavares, Antonio Hernandes Chaves-Neto, Gabriela Alice Fiais, Francilene Lima Agostinho de Souza, Francis Lopes Pacagnelli, Cara Beth Suggett, Giovana Rampazzo Teixeira

**Affiliations:** ^1^Department of Physical Education, São Paulo State University (UNESP), School of Technology and Sciences, Presidente Prudente, São Paulo, Brazil; ^2^Multicenter Graduate Program in Physiological Sciences, SBFis, São Paulo State University (UNESP), Presidente Prudente, SP, Brazil; ^3^Departamento de Fisiología, Facultad de Farmacia, Universidad Complutense de Madrid, Madrid, Spain; ^4^Department of Basic Sciences, School of Dentistry, São Paulo State University (UNESP), Araçatuba São Paulo, Brazil; ^5^Postgraduate Animal Science Program, University of Western São Paulo (UNOESTE), Presidente Prudente, Brazil; ^6^Department of Biomedical Sciences, University of Guelph, Guelph, ON, Canada

## Abstract

Arterial hypertension promotes urological complications by modifying the functional capacity of the urinary bladder. On the other hand, physical exercise has been suggested as a nonpharmacological tool to improve blood pressure regulation. High-intensity interval training (HIIT) can effectively increase peak oxygen consumption, body composition, physical fitness, and health-related characteristics of adults; however, its action on the urinary bladder is little discussed. In the present study, we verified the effect of HIIT on the modulation of the redox state, morphology, and inflammatory and apoptotic processes of the urinary bladder of hypertensive rats. Spontaneously hypertensive rats (SHR) were divided into two groups: SHR sedentary and SHR submitted to HIIT. Arterial hypertension promoted an increase in the plasma redox state, modified the volume of the urinary bladder, and increased collagen deposition in detrusor muscle. It was also possible to identify, in the sedentary SHR group, an increase in inflammatory markers such as IL-6 and TNF-*α* in the urinary bladder, as well as a reduction in BAX expression. However, in the HIIT group, reduced blood pressure levels were observed, together with an improvement in morphology, such as a decrease in collagen deposition. HIIT also regulated the proinflammatory response, promoting increases in IL-10 and BAX expressions and in the number of plasma antioxidant enzymes. The present work highlights the intracellular pathways involved with the oxidative and inflammatory capacity of the urinary bladder and the potential effect of HIIT on the regulation of the urothelium and detrusor muscle of hypertensive rats.

## 1. Introduction

Arterial hypertension, characterized by a sustained increase in blood pressure, affects approximately 1 billion people worldwide, making it one of the leading causes of death from cardiovascular disease [1, 2]. Arterial hypertension is often accompanied by urological complications, including increased urination frequency [3] and decreased bladder capacity [4] and urination volume. Urinary bladder dysfunction is attributed to abnormal neural pathway function and local bladder changes in spontaneously hypertensive rats (SHR) [5]. Another pathophysiological effect related to arterial hypertension is the increased recurrence of urinary tract syndrome (LUTS), which is the main cause of urological morbidities presented in adult men [6], with a recurrent decrease in bladder blood flow (BBF) in SHR in relation to normotensive rats [4].

The high morbidity and negative impacts on the quality of life of individuals affected with arterial hypertension and pathophysiological mechanisms of the urinary bladder are not well elucidated. However, the literature reports pathological progression in the urinary bladder as a complex biochemical pathway involving inflammation and fibrosis [5, 7]. The increased connective tissue in the urinary bladder under blood pressure-demand promotes activation of proinflammatory cytokines [7, 8], interstitial cystitis (IC), and bladder pain syndrome (BPS) [9].

The literature reports several properties of physical activity in different tissues, including the prostate of hypertensive rats [10]. This nonpharmacological therapeutic relevance of the use of physical training could be a starting point for new approaches to the prevention and treatment of urinary tract diseases. Some studies have reported the effects of physical exercise on the urinary bladder. Sanford et al. [11] demonstrated that voluntary aerobic exercise improves voiding function and urinary bladder hyperalgesia in animal models. Current guidelines recommend regular physical activity as a means of preventing arterial hypertension [12], and these exercise recommendations were expanded to include HIIT as an exercise to prevent arterial hypertension [12]. HIIT consists of short-term physical training ranging from 10 seconds to 4 minutes, with an intensity of 80% to 95% of the maximum heart rate, with short pauses for periods of 10 seconds to 3 minutes, whether active or passive [13]. This protocol has been proposed as an alternative to enhance and promote significant changes in the body of patients with cardiovascular diseases, including arterial hypertension [14, 15]. Compared to moderate-intensity aerobic exercise performed on a treadmill, HIIT is a new approach with an impact on blood pressure regulation. Studies in our laboratory have previously demonstrated the role of HIIT in the biological system affected by arterial hypertension. Correia et al. [10] found that arterial hypertension promotes an increase in prostatic lesions and that the HIIT protocol increased the expression of FAS/CD95 and IL-10 proteins, consequently decreasing the incidence of prostatic intraepithelial neoplasia (PIN).

In the present study, we verified the effect of HIIT on the modulation of the redox state, morphology, and inflammatory and apoptotic processes of the urinary bladder of hypertensive rats. The results showed changes in plasmatic redox state, urinary bladder morphology, and inflammatory proteins. The 8-week experimental HIIT protocol was able to efficiently reduce arterial hypertension, leading to reduced impacts on the urinary bladder microenvironment, thus representing an important therapeutic target.

## 2. Materials and Methods

### 2.1. Animals and Ethics Statement

Twenty aged male SHR, 51.4 weeks of age, were obtained from the Central Vivarium of the State University of Campinas. Groups of 3-4 animals were housed in plastic cages with a controlled temperature of 21-23°C, 50-60% humidity, and a 12 : 12 h light/dark cycle. The animals were randomized into 2 experimental groups: spontaneously hypertensive sedentary rats (SHRc, *n* = 10) and spontaneously hypertensive rats submitted to high-intensity interval training (SHR+T, *n* = 10). This study was approved by the Ethics Committee for the Use of Animals (CEUA) of the São Paulo State University, Botucatu Campus, under protocol number 1167/2016. All procedures with animals were performed following the ethical principles for animal research according to the Brazilian College of Animal Experimentation and according to the Guide for the Care and Use of Laboratory Animals from the National Research Council [16].

### 2.2. Maximum Capacity Test

**Figure 1:**
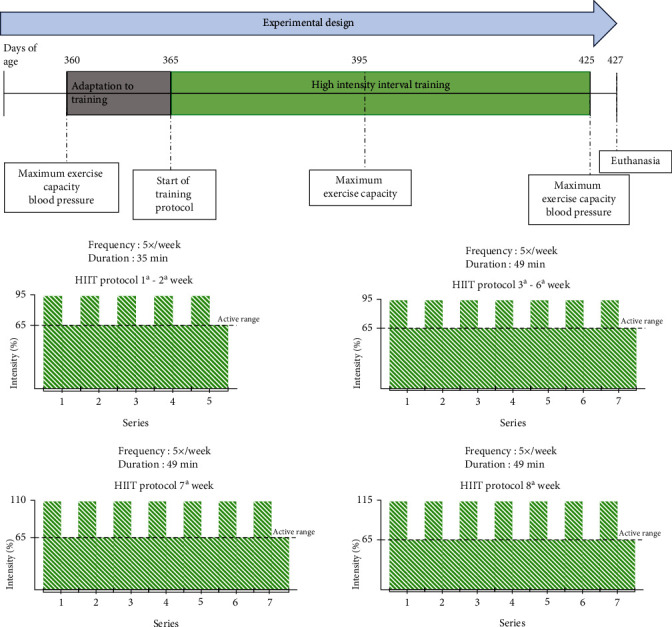
Experimental design of the HIIT protocol for 8 weeks with hypertensive rats (SHR). The animals arrived at 360 days of age, when they passed the battery of tests consisting of maximum physical exercise capacity on a treadmill and measurement of systolic blood pressure. The rats performed 5 days of adaptation to training and the treadmill, starting the HIIT protocol at 365 days of age. At the beginning of the 4th week of training, at 395 days of age, the animals were submitted to a new maximum exercise capacity test, and the training was adjusted. At the end of the 8th week of training, at 425 days of age, the animals were submitted to another battery of tests, identical to the one performed in the initial training period. At 427 days of age, the animals were euthanized by decapitation.

The animals were initially adapted to a treadmill (model TK 1, Inbramed, São Paulo, Brazil) for 5 days (10 min/day). Next, the maximal aerobic capacity test was performed on a treadmill without inclination, with an initial speed of 6 m/min. A load of 3 m/min was applied every 3 min until reaching the maximum speed for each animal. Every 3 minutes when the load was increased, the voluntary exhaustion of the animals was verified. Maximum exhaustion was determined when the rats touched the edge of the treadmill more than 3 times in 1 min or when they could not coordinate their steps [17, 18]. Functional capacity was evaluated according to the total distance covered, calculated considering the speed and maximum time the animal remained in the test. This test was performed at the beginning of the physical training protocol, at the end of the 4th week of the HIIT protocol, and after the final HIIT physical training session [18] (Figure 1).

### 2.3. High-Intensity Interval Training Protocol

The HIIT protocol was developed as previously described [10]. Briefly, after the adaptation period and maximum capacity test, the HIIT protocol on the treadmill began, consisting of physical training sessions with an estimated time of 50 min/day, 5 days a week, for 8 weeks [19]. Each HIIT training session consisted of warm-up, HIIT development, and recovery. We used the overload of 95% of the maximum speed achieved in the exhaustion test for 4 min, interspersed with the overload of 65% of maximum speed for 3 min. During the 1st and 2nd weeks of the experimental protocol, 5 sets were performed. In the 3rd and 4th weeks, the same overload was used with 6 consecutive sets per physical training session. Before starting the 5th week, a new maximal exercise capacity test was performed to reassess the maximal exhaustion velocity, and the training load was readjusted. In the 5th and 6th weeks, the HIIT protocol was adapted [20, 21], starting with a speed of 95% for 4 min, interspersed with 65% for 3 min, in the 7th week at 115%, interspersed with 65% of the maximum speed for 3 min, repeated 7 series [22] (Figure 1).

### 2.4. Systolic Blood Pressure (SBP)

Systolic blood pressure was assessed by plethysmography using the tail-cuff method (Narco Bio-System®, model 7 09-0610, International Biomedical, Inc., USA) before and after the training period [23]. The percentage of SBP variation (Δ) was calculated according to the following formula: [(final value − initial value) ÷ initial value] × 100 [23].

### 2.5. Biochemical Analyses of Plasmatic Redox State

At 427 days of age, 48 hours after the final HIIT session, the animals were anesthetized with ketamine (50 mg/kg/pi) and xylazine (10 mg/kg/pi) and then euthanized by decapitation. The trunk blood was collected and stored in a heparin tube before being centrifuged, and the serum was collected and stored at -20°C. Lipid peroxidation was performed by measuring thiobarbituric acid reactive substances (TBARS) [24]. The number of aldehydes formed was calculated by their extinction coefficient (*ε*_532nm_ = 1.56 × 10^5^ M^−1^ cm^−1^). The total nonenzymatic antioxidant capacity (TAC) was determined spectrophotometrically using the ferric reducing antioxidant power assay (FRAP), as previously described by Benzie and Strain [25]. The results were calculated using a standard curve constructed using different concentrations of ferrous sulfate solutions. Superoxide dismutase (SOD) activity was determined spectrophotometrically (420 nm) by measuring the inhibition of the autoxidation rate of pyrogallol in 50 mmol/L Tris-HCl buffer (pH 8.2) containing 1 mmol/L diethylenetriamine penta-acetic acid. One unit of SOD was defined as the amount of enzyme needed to inhibit the autoxidation rate of pyrogallol by 50% [26]. All values were normalized to total protein measured by the Bradford method [27].

### 2.6. Histological Analysis and Morphometric Analysis

Forty-eight hours after the final HIIT session, the animals were anesthetized with ketamine (50 mg/kg/pi) and xylazine (10 mg/kg/pi) and then euthanized by decapitation. The abdominal-pelvic laparotomy was performed, and the urinary bladder was collected and fixed in Bouin. After 24 hours, the samples were washed in 70% alcohol, dehydrated, diaphonized, embedded in paraplastic, and cut to 5 *μ*m in thickness. Toluidine's blue, hematoxylin and eosin (H&E), and picrosirius red stains were performed. The slides were analyzed and photographed in a Zeiss AxioPhoto photomicroscope with AxioVision Rel. 4.8 software at 400x magnification. For the collagen fiber quantification, the picrosirius red technique was used, with a magnification of 400x. We used ImageJ software, version 1.50i (National Institutes of Health, Bethesda, MD, USA) for quantification with Masson's trichrome plugin. The percentage of marking was quantified for each image, and the values of the amount of pigmentation of the cells were given as a percentage of the marked area [28]. The mast cells were analyzed using the toluidine blue technique and measured by cells/mm^2^. The photos used for this were magnified 400x, with an area of 0.68mm^2^, and 12 photos/area were obtained per animal [29].

For the morphometric evaluation, three cross-sections of the urinary bladder were obtained per animal, with a 12x magnification (ZEISS SteREO Discovery V12 Microscope). For quantification, the ImageJ 1.5 program was used, with the help of the line selection tool, where 5 transverse lines are drawn per section, to measure the thickness of the wall or urothelium. This totaled 15 measurements per animal. The thickness of each measurement was expressed in millimeters (mm) or micrometers (*μ*m). For volume analysis, the total width of the urinary bladder was measured, and the single-plane ellipsoid model (SP) method was used [30] with the following equation for volume (*V*):

(1) $$\begin{array}{c} V=0.85\times \frac{A2}{W}, \end{array}$$

where *V* is the volume, *A* is the area, and *W* is the width.

To obtain the area, the following equation was used:

(2) $$\begin{array}{c} A=R1\times R2\times \pi , \end{array}$$

where *A* is the area, *R*1 is the transverse radius, and *R*2 is the longitudinal radius.

### 2.7. Bladder Inflammation Index (BII)

The bladder inflammation index was measured based on leukocyte infiltration in the lamina propria as previously described elsewhere [31], considering the infiltration of leukocytes in the lamina propria. Leukocyte infiltration was evaluated using H&E staining. Scores for leukocyte infiltration were obtained from three cross-sections of distinct portions of the urinary bladder of each animal, in nine animals per group. The total number of leukocytes was divided by the area in mm^2^ of each portion, to identify the quantity/mm^2^. Leukocyte infiltration (polymorphonuclear (PMN) and mononuclear cells) in each section was evaluated using the following scores: 0 = no leukocytes found per mm^2^; 1 = mild infiltration or less than 20 leukocytes found per mm^2^; 2 = moderate infiltration, between 20 and 45 leukocytes found per mm^2^; and 3 = severe infiltration, equal to or greater than 45 leukocytes found per mm^2^.

### 2.8. Immunohistochemical Analyses

The slides were placed in the microwave at 700-800 W, and the sections were incubated in sodium citrate buffer (0.01 M, pH 6.0). Then, to block endogenous peroxidase, the sections were submitted to a solution of hydrogen peroxide 3% and methanol. To block unspecific reactions, we incubated the slides in a bovine serum albumin (BSA 3%) solution. In the next step, the sections were subjected to a reaction with the primary antibodies BCL-2 (1 : 100, N-19, sc-492, Santa Cruz Biotechnology®), BAX ((1 : 100, P-19, sc-526, Santa Cruz Biotechnology®), IL-6 (1 : 100, E-4, sc-28343, Santa Cruz Biotechnology®), IL-10 (1 : 100, NYRm, sc-733309, Santa Cruz Biotechnology®), and TNF-*α* (1 : 100, 52B83, sc-527, Santa Cruz Biotechnology®) and incubated overnight. The sections were incubated with anti-rabbit and/or anti-goat HRP secondary antibody conjugated at a 1 : 200 dilution in bovine serum albumin (BSA 1%). The slides were revealed with diaminobenzidine (DAB) chromogen and counterstained with hematoxylin.

The intensity of BCL-2, BAX, IL-6, IL-10, and TNF-*α* immunoreactivity antigens was examined in 12 fields per animal using ImageJ software, version 1.50i (National Institutes of Health, Bethesda, MD, USA). For DAB quantification, the percentage of marking was quantified for each image, and the cell immunopositivity was given as the percentage of the marked area [28, 32].

## 3. Statistical Analysis

The Shapiro-Wilk normality test was performed to verify all datasets analyzed in the Gaussian distribution model. The results are presented as mean and standard deviation. Comparisons were performed using Student's *t*-test for unpaired samples. To compare the mean blood pressure variation between the initial period (baseline) and after 8 weeks (final) of the experimental protocol, the variation delta (Δ%) was used through the calculation [(final value − initial value) ÷ initial value] × 100. All statistical analyses were performed in GraphPad Prism 8 software, adopting a significance of *p* < 0.05.

## 4. Results

### 4.1. HIIT Attenuates Body Weight Loss in SHR

**Figure 2:**
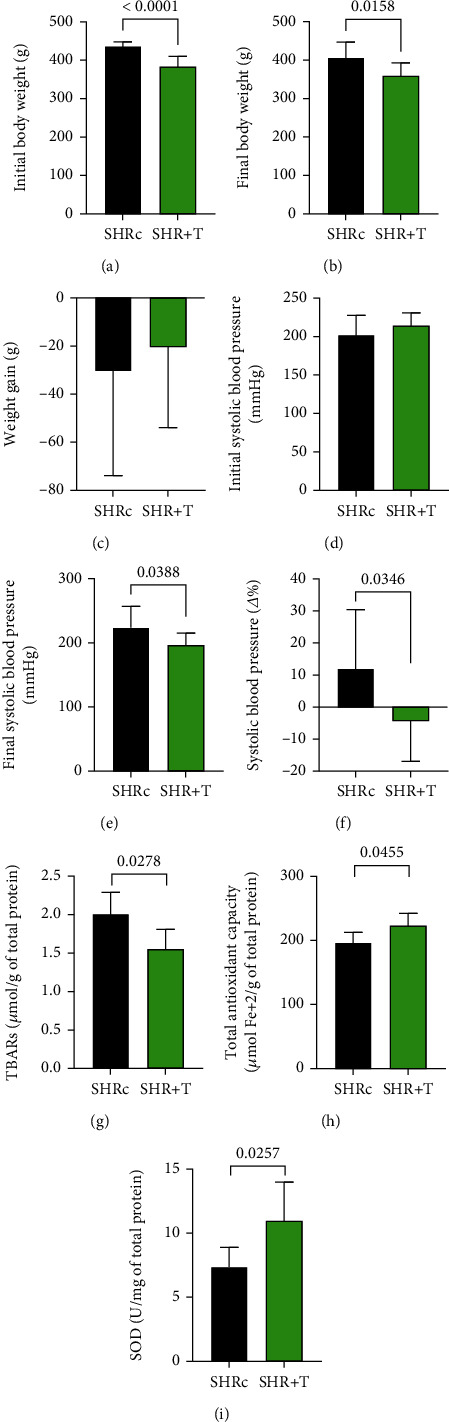
Graphic representation of body weight, blood pressure levels, and redox state in SHRc and SHR+T animals. Graphs (a) to (c) represent the body weight values of the animals. Graph (a) refers to the initial body weight, graph (b) refers to the final body weight after 8 weeks of intervention with HIIT, and graph (c) refers to the Δ% variation in body weight. Graphs (d) to (f) represent the pressure values of the animals. Graph (d) refers to the baseline pressure levels of the animals, graph (e) refers to the resulting pressure levels after 8 weeks of intervention with HIIT, and graph (f) refers to the Δ% change in blood pressure. Graphs (g) to (i) represent the plasma redox status. Graph (g) refers to oxidative damage data obtained by TBARS, graph (h) refers to total nonenzymatic antioxidant capacity data obtained through FRAP, and graph (i) refers to SOD activity.

Figure 2 presents the body weight data of the SHR animals. Sedentary hypertensive animals showed an average reduction of -30.44 g in body weight during the experimental period (Figure 2). During the 8 weeks of the HIIT protocol, the hypertensive rats showed a reduction of -20.44 g in body weight, evidencing the effect of physical exercise.

### 4.2. HIIT Lowered Systolic Blood Pressure

To identify the effects of AH on the urinary bladder and whether HIIT could minimize its effects, we measured blood pressure levels at baseline and after the physical training protocol. At baseline, the groups presented high systolic pressure values, characterizing arterial hypertension, with no significant difference between the groups (*p* = 0.225; Figure 2(a)). After 8 weeks of the HIIT protocol, the SHR+T group presented significantly decreased systolic blood pressure values when compared to SHR-c (*p* = 0.0388; Figure 2(b)). The delta value was calculated to check the variation (Δ%) of blood pressure between baseline and across the 8 weeks, and it was possible to observe the efficiency of the SHR+T group in reducing systolic blood pressure when compared to the SHR group (*p* = 0.0346; Figure 2(c)).

### 4.3. HIIT Reduces Oxidative Stress and Increases Antioxidant Activity

Lipid peroxidation occurs when reactive oxygen species (ROS) are generated, resulting in the accumulation of so-called thiobarbituric acid reactive substances, or TBARS [33]. Chronic arterial hypertension leads to vascular damage and increased ROS. Moreover, ROS may underlie urinary bladder dysfunction [34]. Thus, we sought to investigate whether chronic arterial hypertension leads to free radical generation in the urinary bladder and the potential effects of HIIT. We analyzed the total oxidative activity by TBARS and found that total oxidative activity was significantly reduced in the SHR+T group compared to the SHR group (*p* = 0.0278; Figure 2(d)).

To assess the plasma TAC induced by HIIT, we used the FRAP method. The HIIT protocol in the SHR+T group raised the levels of TAC compared to the SHRc group (*p* = 0.0455; Figure 2(e)). There are essential antioxidant enzymes in the cell, one of which is superoxide dismutase (SOD) activity. Corroborating the TAC data, the SHR-T group presented higher SOD values when compared to the SHRc group (*p* = 0.0257; Figure 2(f)). Given our findings, it was possible to conclude that the 8-week HIIT protocol reduced the oxidant levels shown by the TBARS in SHR rats, concomitantly increasing the enzymatic and nonenzymatic antioxidant effects with the increases in SOD and TAC, respectively.

### 4.4. HIIT Training Improves the Urinary Bladder Morphology and Structure

**Figure 3:**
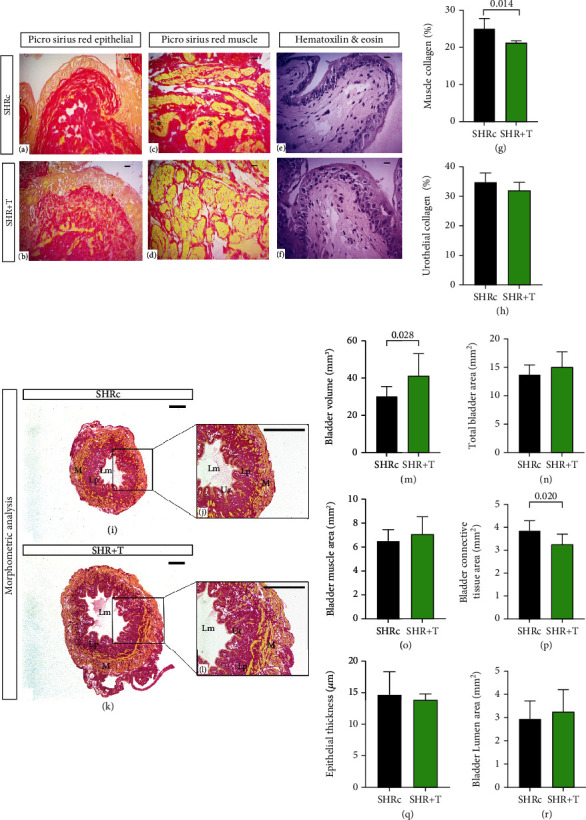
Histological and morphometric analysis: (a, b) picrosirius red epithelial; graph (h) shows the amount of collagen found in the urinary bladder epithelium; (c, d) picrosirius red muscular; graph (g) shows the amount of collagen found in the detrusor muscle of the urinary bladder; (e, f) hematoxylin and eosin staining (H&E); (i–l) data of morphometric analysis; graph (m) refers to the bladder volume; (n) total bladder area; (o) bladder muscle area; (p) bladder connective tissue area; (q) bladder epithelium thickness bladder; (r) luminal area of the bladder. (a–f) Bar = 20 *μ*m, 400x resolution; (i, k) bar = 1 mm, 12x resolution; (j, l) bar = 1 mm, 31x resolution. The unpaired *t*-test was used for comparisons between groups. Data are expressed as mean ± SD.

Convincing evidence has linked obstructive bladder dysfunction and bladder dysfunction with ischemia generated by arterial hypertension, free radical generation, and oxidative damage to smooth muscle and mucosa [35]. We sought to investigate whether chronic hypertension leads to morphological and structural urinary bladder changes in SHR and the potential effects of HIIT. First, we found that SHR presented more collagen deposition in the submucosal layer (Figures 3(a) and 3(b)) and detrusor muscle (Figures 3(c) and 3(d)) and more abundant connective tissue (Figure 3(p)) compared to the SHR+T group (Figures 3(b) and 3(g), *p* = 0.165 vs. control; Figures 3(d) and 3(h), *p* = 0.014 vs. control; and Figure 3(p), *p* = 0.020 vs. control). Quantitative analysis of the detrusor muscle revealed that the SHR+T group has thicker muscle area when compared to the SHRc group (*p* = 0.372; Figure 3(o)). On the other hand, the SHR+T group showed thick mucosa when compared to the SHRc group (*p* = 0.623; Figure 3(q)). With the increase in the muscular layer and a reduction in the mucosa, an increase in bladder lumen area was observed in the SHT+T group (Figures 3(j), 3(k), and 3(r); *p* = 0.413 vs. control). Moreover, the SHR+T group had higher total urinary bladder volume (Figure 3(m), *p* = 0.0283 vs. control) and total bladder area (Figure 3(n), *p* = 0.251 vs. control) when compared with the SHRc group. Our findings show that arterial hypertension reduced functionality of the bladder in the SHRc group. The 8-week HIIT protocol led to an increase in the volumes and areas of the urinary bladder, improving their functionality, leading to increases in the volume of the detrusor muscle and lumen and reductions in intramuscular and submucosal collagen deposition.

### 4.5. HIIT Decreases Inflammatory Profile of Urinary Bladder in SHR

**Figure 4:**
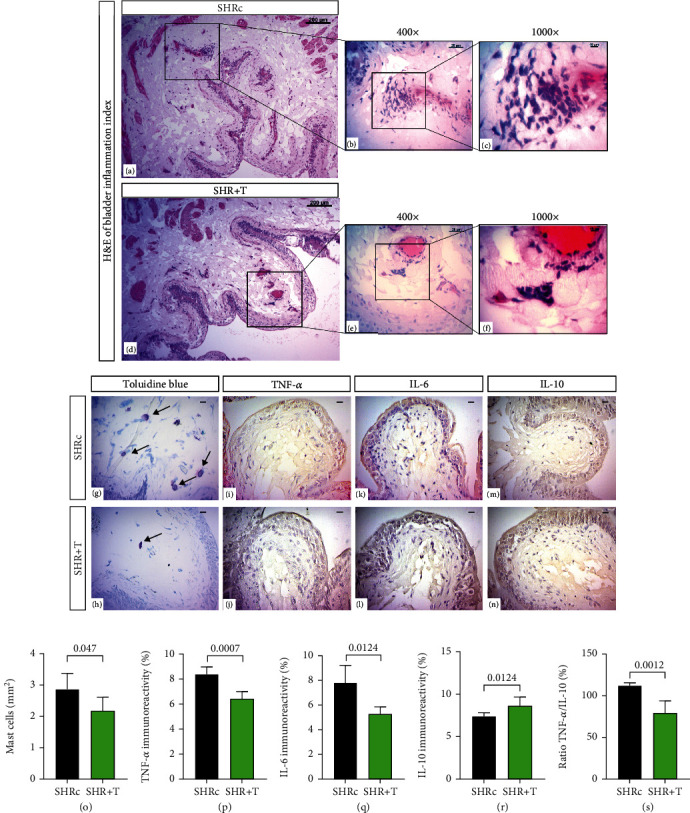
Histological analysis of bladder inflammation score and immunohistochemistry: (a–c) H&E technique with inflammatory infiltrate in the SHRc group; (d–f) H&E technique with inflammatory infiltrate in the SHR+T group; (g, h) toluidine's blue; (i, j) related to TNF-*α* labeling; (k, l) related to IL-6 expression; (m, n) related to IL-10 expression. Graph (o) refers the Mast cell count; (p) indicates the expression of TNF-*α*; (q) indicates the IL-6; (r) indicates the expression of IL-10; (s) indicates the TNF-*α*/IL-10 ratio. (a, d) Bar = 200 *μ*m, 100x resolution; (b, e) bar = 20 *μ*m, 400x resolution; (c, f) bar = 10 *μ*m, 1000x resolution; (g, n) bar = 20 *μ*m, 400x resolution. The unpaired *t*-test was used for comparisons between groups. Data are expressed as mean ± SD.

Growth mediators and cytokines are potent modulators of cellular proliferation, morphology, and function [9]. In our study, when analyzing the bladder inflammation score, the samples from the SHR+T group (21.44 ± 6.38) presented leukocyte infiltration with a mild degree of infiltration, presenting a lower degree of infiltration when compared to the SHRc group (30.13 ± 6.24) (Table 1 and Figure 4) which showed a moderate degree of inflammation due to greater leukocyte infiltration. The HIIT protocol reduced the number of mast cells (*p* = 0.047; Figures 4(g)–4(i) and 4(p)). Regarding the expression of cytokines, the expression of TNF-*α* decreased (*p* = 0.0007; Figures 4(j), 4(k), and 4(q)) and the expression of IL-6 decreased when compared to the group SHRc (*p* = 0.012; Figures 4(l), 4(m), and 4(r)). Submucosal IL-10 expression was lower in the SHRc group, but after the bladder HIIT protocol, IL-10 expression significantly increased in the submucosal connective tissue and between smooth muscle bundles in the SHR+T group (*p* = 0.0414; Figures 4(n), 4(o), and 4(s)). The TNF-*α*/IL-10 ratio was reduced as predicted due to the anti-inflammatory profile of physical exercise; the HIIT protocol (SHR+T) significantly reduced the inflammatory profile compared to SHRc (*p* = 0.0012; Figure 4(t)). Therefore, our data suggest that the 8-week HIIT protocol was efficient to reduce inflammation and improve the deleterious effects generated by arterial hypertension in the bladder microenvironment.

### 4.6. HIIT Training Induced Urinary Bladder Apoptosis of the Urothelium in SHR

**Figure 5:**
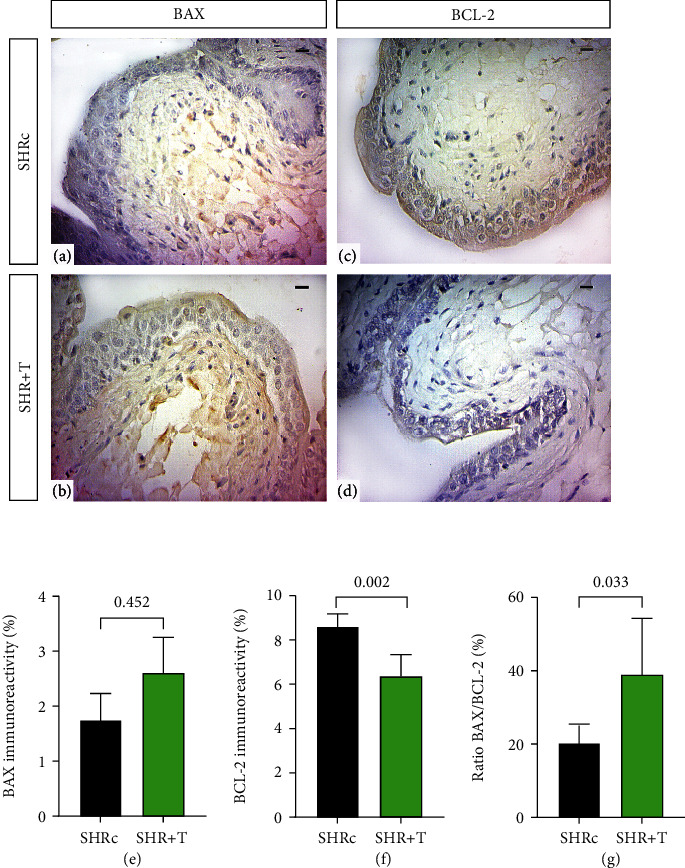
Immunohistochemistry data of the urinary bladder of hypertensive rats trained for 8 weeks: (a, b) related to BAX immunostaining; (c, d) related to BCL-2 expression. Graph (e) indicates the BAX tag; (f) indicates the expression of BCL-2; (g) indicates the BAX/BCL-2 ratio. The unpaired *t*-test was used for comparisons between groups. Bar = 20 *μ*m, 400x resolution. Data are expressed as mean ± SD.

Next, to understand the influence of HIIT, we investigated whether the protein levels of apoptosis regulators (BCL-2 and BAX) and the BAX/BCL-2 expression ratio in the bladder of SHR animals could serve as clinical outcome parameters. The proteins BAX (proapoptotic) and BCL-2 (antiapoptotic) were verified immunohistochemically. The SHR+T group submitted to the HIIT protocol showed a significant increase in BAX expression levels compared to the SHRc group (*p* = 0.0452; Figures 5(a), 5(b), and 5(e)). From another perspective, the sedentary SHRc group showed increased expression of BCL-2 in comparison to the SHR+T group (*p* = 0.002; Figures 5(c), 5(d), and 5(f)). The BAX/BCL-2 ratio was decreased in the SHRc group, perceived as a reflection of the imbalance generated by arterial hypertension; thus, the SHR+T group presented increases in this ratio through regulation of the imbalance generated by hypertension (*p* = 0.033; Figure 5(g)). Taking these results together, we can conclude that the HIIT protocol generates regulation of the apoptotic pathway in the urinary bladder, decreasing the reduction in the epithelium generated by arterial hypertension.

## 5. Discussion

In the current study, we found that HIIT minimized the effects caused by arterial hypertension on the urinary bladder. Thus, the main findings were as follows: (1) HIIT reduced systolic blood pressure and weight loss, decreased plasma oxidative stress, and increased antioxidant capacity; (2) urinary dysfunction caused by arterial hypertension reduced the total volume of the bladder and increased the collagen area in the muscle layer; (3) HIIT increased IL-10 levels and reduced levels of inflammatory and antiapoptotic cytokines in the urinary bladder; and (4) HIIT induced an increase in the volume, inducing collagen reduction and higher levels of BAX protein. Taken together, our data show that arterial hypertension can modify the expression of proteins related to cell survival, altering urinary functionality (Figure 5). On the other hand, HIIT significantly influenced the apoptotic balance of the urinary bladder. As far as we know, this is the first report of morphological alterations in the bladder resulting from arterial hypertension and positive modulations of high-intensity physical exercise.

Arterial hypertension induces blood flow-related urinary bladder dysfunction, including a reduction in bladder capacity and voiding volume [36, 37]. Male patients with hypertension presented more severe LUTS than normotensive individuals [38]. Thus, the findings suggest that hypertension results in mechanical and functional problems in the urinary bladder [39]. In the current study, we observed that arterial hypertension leads to morphological alterations in the urinary bladder, resulting in lower urinary volume, larger collagen area, and smaller lumen, followed by a decrease in detrusor muscle area. Herein, long-term HIIT training was effective in improving urinary bladder volume, as well as reducing collagen content and increasing muscle area. Thus, we assume that a regulatory mechanism for the blood flow disorder was reestablished with increasing intensity of physical training. HIIT promoted a reduction in blood pressure after 8 weeks of physical training. However, the exact mechanisms underlying the observed changes promoted by HIIT and hypertension in relation to urinary bladder function and structure are still unclear.

To explore the molecular events of oxidative stress associated with HIIT and hypertension, we analyzed the plasma redox status. Oxidative stress plays a critical role in the pathogenesis of arterial hypertension [40] and SHR present markers of increased oxidative stress and bladder dysfunction related to hypertension [41]. In our study, we investigated the impact of HIIT on antioxidant enzymes and ROS in SHR [42, 43]. The increased levels of TBARS are correlated with increased lipid peroxidation damage to cell components, and the concentration of TBARS has a positive correlation with the development of malignant lesions in the urothelium [44]. The oxidative damage, as assessed by the levels of TBARS, was increased in the plasma of SHR. For the first time, we demonstrated that HIIT training can reduce oxidative damage and consequently significantly increase SOD activity and TAC levels. Antioxidant mechanisms such as SOD represent the primary cellular defense against superoxide radicals [45]. Lipid peroxidation serves as a marker of prolonged oxidative endothelial damage, leading to the development of hypertension and atherosclerosis [46]. Our data clearly indicate that HIIT can beneficially modulate oxidant-antioxidant imbalance in the urinary bladder of SHR.

Bladder modifications, such as the recruitment of inflammatory cells, are a key issue in the induction of hypertension-mediated inflammation. Mast cells play an important role in inflammatory diseases and are increased in patients with interstitial cystitis/bladder pain syndrome [47]. It is known that long-term arterial hypertension increases the total number of mast cells [48], induces degranulation of the bladder, and increases the production of proinflammatory cytokines including IFN-*γ*, TNF-*α*, IL-1, and IL-17, which contribute to the pathogenesis of inflammation [49]. In our study, we also found that arterial hypertension promoted an increase in Il-6 and TNF-*α* in bladder tissue. IL-10 is an anti-inflammatory cytokine and is an important cytokine within the group of Th2-inducing cytokines in the immune response to the urinary bladder [50]. On the other hand, HIIT downregulated TNF-*α* and IL-6 and upregulated IL-10 expression, subsequently attenuating hypertension-induced inflammation.

It is known that the tissue alterations caused by arterial hypertension are due to the sympathetic discharge that generates hyperactivity of the urinary bladder. This generates an increase in oxidative stress and inflammation, thus inducing tension overload [51, 52]. We evaluated the intrinsic pathway of apoptosis and noticed that arterial hypertension generated an increase in the antiapoptotic protein BCL-2 and a decrease in the proapoptotic protein BAX and consequently considerably decreased the BAX/BCL-2 ratio. The relative increase in BCL-2 may be related to the progression of increased survival of urinary bladder cells, mainly epithelial cells [53]. A decreased BAX/BCL-2 ratio indicates cell survival of possible malignant or injured cells when under stress [54]. Our group demonstrated that moderate or intense resistance or aerobic exercise promotes alteration in urogenital apoptosis, increasing BAX and reducing BCL-2, even if the animals are submitted to high-fat diets or have arterial hypertension [10, 28, 29, 32]. In the current study, we confirmed that the HIIT protocol promoted a reduction in BCL-2 and increased BAX expression, thereby altering the BAX/BCL-2 ratio. Thus, intrinsically regulating the apoptosis of urinary bladder cells reduces the recurrence of cell proliferation that may be damaged by arterial hypertension, maintaining tissue homeostasis.

**Figure 6:**
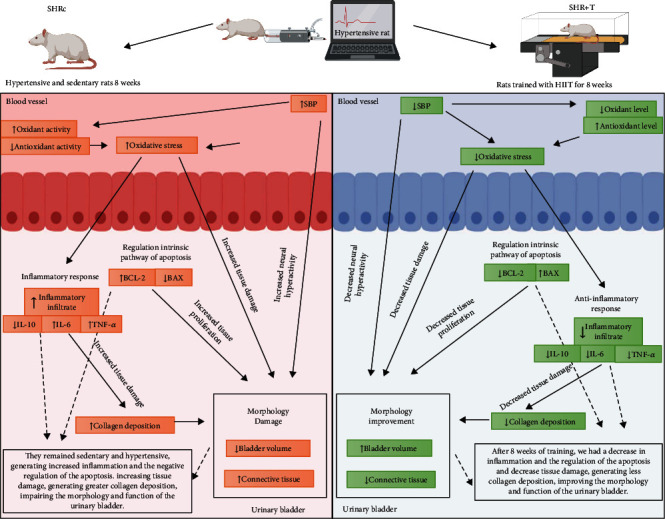
In the urinary bladder of animals in the SHRc group, we found an increase in inflammatory responses resulting from the increase in proteins such as IL-6 and TNF-*α*. During the hypertensive state, with increased blood pressure, the shear stress, sympathetic discharge, and increased angiotensin II generate vascular damage, increasing blood oxidative stress, in addition to arterial hypertension itself increasing oxidant molecules and decreasing antioxidant molecules, corroborating the increase in blood pressure and oxidative stress. These stressors lead to increases in the tissue inflammatory process in the urinary bladder, together with the neural hyperactivity resulting from hypertension, increasing tissue damage in the bladder and collagen deposition, mainly in the region of the detrusor muscle, which decreases and compromises the functionality of the urinary bladder. Another factor modulated by hypertension found in our SHRc animals was the intrinsic pathway of apoptosis, for tissue increase, decreasing the capacity and volume of the urinary bladder, increasing connective tissue, and decreasing the lumen of the UB. In contrast to these results, after the 8-week HIIT protocol, the data demonstrate an increase in IL-10 cytokine expression and low expression of IL-6 and TNF-*α*. Due to physical training, there is a decrease in blood pressure, which reduces shear stress and sympathetic discharge, thus resulting in a decrease in oxidative stress in the animals of the trained group, in this way, helping to reduce tissue inflammation, thus decreasing collagen deposition in the bladder and improving the function and capacity of the organ. Furthermore, HIIT improved the BAX and BAX/BCL-2 ratio when compared to the SHRc group, indicating regulation of the intrinsic pathway of apoptosis, controlling the tissue balance of the urinary bladder, and repairing the urinary bladder morphology and function, as a result of increased volume, muscle, and luminal area. Finally, these data demonstrate the influence of the HIIT protocol on AH, improving the vesical microenvironment of the animals' bladder.

Overall, our data suggest that arterial hypertension exerts distinct changes in circulating ROS and antioxidant enzymes. In addition, high blood pressure affects inflammation in the urothelium and submucosa. However, HIIT improved activation of antioxidant enzymes and reduced inflammation and may increase apoptosis. To the best of our knowledge, this is the first comprehensive characterization of the altered histology and inflammatory pathway together with HIIT and arterial hypertension in the bladder. Given the significant similarity between the physiology of hypertensive humans and rodents, these findings have high translational value. Finally, we can infer that HIIT can reduce the clinical risk of urinary bladder alterations in vivo, even in hypertensive rats (Figure 6).

## 6. Conclusion

In conclusion, arterial hypertension promoted volume reduction and increased collagen deposition in the urinary bladder, which are related to bladder stiffening and functionality. Inflammatory cytokines and oxidative stress increased cellular damage. On the other hand, our experimental design with an 8-week HIIT protocol was enough to reduce the harm caused by arterial hypertension. In conclusion, it was possible to demonstrate that the HIIT training protocol promoted positive morphological alterations in the urinary bladder microenvironment of SHR rats. This indicates that HIIT could represent a nonpharmacological treatment for the urinary bladder in arterial hypertension.

## Figures and Tables

**Table 1:** Bladder inflammatory index data from hypertensive and HIIT-trained rats.

Bladder inflammation index (BII)						
	0	1	2	3	Media cell (mm^2^)	*p* value
SHRc (*n* = 8)	0	0	8	0	30.13	0.0247^∗^
SHR+T (*n* = 9)	0	6	3	0	21.44	

Legend: urinary bladder inflammatory index in SHRc and SHR+T animals, score 0 = no leukocytes found per mm^2^; 1 = mild infiltration or less than 20 leukocytes found per mm^2^; 2 = moderate infiltration, between 20 and 45 leukocytes found per mm^2^; and 3 = severe infiltration, equal to or greater than 45 leukocytes found per mm^2^. The unpaired *t*-test was used for comparison between groups. Data are expressed as mean ± SD. ^∗^*p* value of 5% for significance or *p* < 0.05.

## Data Availability

The datasets used and/or analyzed during the present study come from procedures with animals (approved by the Ethics Committee for the Use of Animals-CEUA of the São Paulo State University, Botucatu Campus, under the protocol number 1167/201), and the results can be requested without restrictions from the corresponding author. There are any restrictions on data access.

## Abbreviations

Δ: Delta
*μ*m: Micrometers
A: Area
AH: Arterial hypertension
Akt: Serine/threonine kinase
Ang II: Angiotensin II
BAX: Bcl2-associated X
BBF: Bladder blood flow
BCL-2: B-cell lymphoma 2
BOO: Bladder outlet obstruction
BSA: Bovine serum albumin
BPS: Bladder pain syndrome
CAPES: Coordenação De Aperfeiçoamento De Pessoal De Nível Superior
CAT: Catalase
CEUA: Ethics Committee on the Use Of Animals
CNPq: National Council for Scientific and Technological Development
COBEA: Ethics Committee on the use of animals
CVDs: Cardiovascular diseases
DAB: 3,3′-Diaminobenzidine
DNA: Deoxyribonucleic acid
FAPESP: São Paulo State Research Support Foundation
FAS/CD95: Fas cell surface death/cluster of differentiation 95
FRAP: Ferric reducing antioxidant power assay
H&E: Hematoxylin and eosin
HIIT: High-intensity interval training
IARC: International agency for research on cancer
IC: Interstitial cystitis
IGF-1: Insulin-like growth factor-1
IL-10: Interleukin 10
IL-1*α*: Factor interleukin-1 alpha
IL-6: Interleukin 6
LUTS: Lower urinary tract syndrome
mm: Millimeters
mTOR: Mammalian target of rapamycin
NADPH: Nicotinamide adenine dinucleotide phosphate
NGF: Nerve growth factor
NO: Nitric oxide
PIN: Prostatic intraepithelial neoplasia
R1: Transverse radius
R2: Longitudinal radius
ROS: Reactive oxygen species
SBP: Systolic blood pressure
SHR: Spontaneously hypertensive rats
SHR+T: Spontaneously hypertensive rats+training
SHRc: Spontaneously hypertensive rat control
SOD: Superoxide dismutase
TAC: Total antioxidant capacity
TBARS: Thiobarbituric acid reactive substances
TGF-*β*: Transforming growth factor-beta
TNF-*α*: Tumor necrosis factor alpha
UB: Urinary bladder
UNICAMP: State University of Campinas
V: Volume
W: Width.
